# Comparison of C:N:P Stoichiometry in the Plant–Litter–Soil System Between Poplar and Elm Plantations in the Horqin Sandy Land, China

**DOI:** 10.3389/fpls.2021.655517

**Published:** 2021-04-26

**Authors:** Kai Wang, Risheng Zhang, Lining Song, Tao Yan, Enhang Na

**Affiliations:** ^1^College of Environmental Sciences and Engineering, Liaoning Technical University, Fuxin, China; ^2^Liaoning Institute of Sandy Land Control and Utilization, Fuxin, China; ^3^Institute of Applied Ecology, Chinese Academy of Sciences, Shenyang, China; ^4^College of Pastoral Agriculture Science and Technology, Lanzhou University, Lanzhou, China

**Keywords:** desertified region, forest decline, plant–litter–soil system, stoichiometric homeostasis, tree species selection for afforestation

## Abstract

Afforestation is among the most effective means of preventing and controlling desertification. Silver poplar (*Populus alba*) is commonly planted tree species for afforestation of the Horqin Sandy Land of China. However, this species has exhibited some drawbacks such as top shoot dieback, premature senescence and mortality, and soil and ecosystems degradation. In contrast, Siberian elm (*Ulmus pumila*) rarely experiences these problems in the same regions. Ecological stoichiometry plays a vital role in exploring ecological processes and nutrient cycle relationships in plant–litter–soil systems. To explore the differences in the carbon (C), nitrogen (N), and phosphorus (P) balance, the stoichiometry characteristics and stoichiometric homeostasis in elm and poplar plantations in the Horqin Sandy Land, we measured C, N, and P concentrations in leaves, branches, roots, litter, and soils and analyzed N and P resorption efficiencies in the two plantations. The results showed that soil C and N concentrations, C:P, and N:P were greater in the elm plantation than in the poplar plantation. The leaf and root C:P and N:P during summer and litter N and P concentrations were greater, whereas N and P resorption efficiencies were lower, in the elm plantation than in the poplar plantation. Generally, elm exhibited greater N:P homeostasis than poplar. N and N:P homeostasis were greater in roots than in leaves and branches in the elm plantation, but they varied with soil N concentration and N:P in the poplar plantation. These findings indicate that poplar exhibited more developed internal nutrient conservation and allocation strategies but poor nutrient accumulation in soil, which may contribute to degradation of poplar plantation. In contrast, elm tended to return more nutrients to the soil, showing an improved nutrient cycle in the plant–litter–soil system and increased soil C and N accumulation in the elm plantation. Therefore, compared with poplar, elm may be a more suitable afforestation tree species for the Horqin Sandy Land, in terms of promoting the accumulation of soil nutrients and enhancing nutrient cycling in the plant–litter–soil system.

## Introduction

Desertification is a serious global environmental problem, with substantial effects on the survival and development of some plant and animal species, human wellbeing and society, and ecosystem stability maintenance (Sterk et al., 2016; Capozzi et al., 2018). The Horqin Sandy Land (42°41′–45°15′ N, 118°35′–123°30′ E) is among the most seriously desertified and ecologically fragile regions in China’s agro-pastoral ecotone; originally a prairie, this sandy land developed due to climate change and human disturbances such as overgrazing, non-manure cropping, and arbitrary land use and management (Zeng and Jiang, 2006). During the desertification process, an estimated 90% of carbon (C) and 86% of nitrogen (N) were lost from the ecosystem (Li et al., 2006). To combat and control desertification, afforestation programs have been launched since the 1970s, including the Three-North Shelterbelt Program, the Grain for Green Project and the Conversion of Cropland to Forest and Grassland Program (Wang, 2014; Bai et al., 2019; Chu et al., 2019).

These afforestation programs selected tree species with drought tolerance, rapid growth, and high timber production traits (Song et al., 2020). Silver poplar (*Populus alba*) is among the most common afforestation tree species due to its relatively high initial growth and seedling survival rates (Lindroth and Clair, 2013), and it has been planted as a monoculture in large areas for wind speed reduction, sand fixation, and soil and water conservation (Zhao et al., 2008; Ahmed et al., 2020). However, these large-scale poplar plantations have many drawbacks including top shoot dieback, tree premature senescence and mortality, and soil and ecosystem degradation (Wang et al., 2017; Zhou et al., 2020). In contrast, Siberian elm (*Ulmus pumila*) rarely exhibits these problems in either natural or plantation forests in the same regions (Zhao et al., 2010). Nevertheless, elm is seldom planted for afforestation in the sandy land due to its slow growth rate and production of crooked trunks, which limit its economic value (Wang et al., 2017). Vegetation conversions after afforestation often involve tremendous changes in plant and soil nutrient concentrations, biomass production, soil quality, and nutrient cycling processes, which profoundly influence the stability and sustainable development of ecosystems (Zhao et al., 2008; Liu et al., 2018; Luo et al., 2020). Therefore, it is necessary to explore the differences in plant and soil nutrients and their interactions between poplar and elm plantations to determine which is more suitable for afforestation.

C, N and phosphorus (P) are major macroelements necessary for life; their cycling in plant–litter–soil systems has substantial effects on the function and stability of ecosystems (Mulder and Elser, 2009). Soil C, N, and P greatly affect plant growth and development and are simultaneously affected by organic matter, litter, and microbes (Sinsabaugh et al., 2008). Litter stores nutrients and returns them to soil; these processes are restricted by nutrient resorption, which contributes to optimal nutrient use efficiency by plants (Deng et al., 2019). Plants adjust their growth rates by coordinating the ratios of C, N, and P and allocating nutrients among different organs to adapt to soil nutrient conditions (Daufresne and Loreau, 2001; Fang et al., 2019). Thus, the balances and interactions of C, N, and P are highly complex in plant–litter–soil systems (Manzoni et al., 2008; Yang et al., 2019).

Ecological stoichiometry, which is used to evaluate the balances of energy and chemical elements in ecosystems, is a powerful tool for understanding ecological processes and relationships among element cycles in plant–litter–soil systems (Elser et al., 2010). Plant C:N:P stoichiometry reflects the efficiency of plants nutrient use (Niklas and Cobb, 2005) and can be used to determine nutrient limitations for growth (Koerselman and Meuleman, 1996). Soil C:N:P stoichiometry reflects soil fertility and nutrient availability and regulates plant growth and the nutrient state (Bui and Henderson, 2013). Stoichiometric homeostasis, a central concept of ecological stoichiometry, is defined as the ability of plants to maintain a relatively stable nutrient composition, regardless of soil nutrients changes (Sterner and Elser, 2002). Higher stoichiometric homeostasis in plants contributes to sustaining the functions and stability of the ecosystem (Yu et al., 2010). When soil nutrients limit plant growth, plants can respond via multiple physiological mechanisms to improve the internal availability and use efficiency of the limiting nutrient, thereby maintaining stability and its associated functions in the body at the limited nutrient level (Hessen et al., 2004). These mechanisms of nutrient conservation in plants include excreting hydrogen ions or enzymes into the soil (Yuan et al., 2019), altering the allocation of photosynthetic products and nutrients among different organs (Peng et al., 2016), and remobilizing nutrients from senescent to other organs before senescence (i.e., nutrient resorption) (Kobe et al., 2005; Wang et al., 2020). Therefore, evaluating C:N:P stoichiometry and stoichiometric homeostasis in a plant–litter–soil system could improve our understanding of plant adaptive mechanisms, nutrient cycles and ecosystem stability.

In this study, we examined seasonal variations in C, N, and P concentrations and their ratios in the leaves, branches, roots, and soils of poplar and elm trees in plantations in the Horqin Sandy Land, China, throughout the growing season. We quantified C, N, and P stoichiometry in leaf litter and analyzed the nutrient resorption efficiency (NuRE) and stoichiometric homeostasis of both tree species. One objective of this study was to determine whether the soil C, N, and P concentrations are lower in the poplar plantation than in the elm plantation, since poplar has a higher growth rate and greater biomass (Zhao et al., 2010) and therefore is expected to consume more nutrients than elm. We also aimed to determine whether poplar has lower plant N and P concentrations than those of elm, due to N and P dilution in response to higher poplar growth rates (Zhao et al., 2010), and whether elm, a native tree species, exhibits greater stoichiometric homeostasis than does poplar, an exotic tree species, since native tree species tend to adapt better than exotic species in local environments (Song et al., 2020).

## Materials and Methods

### Study Site

This study was conducted at the Zhanggutai Experimental Base of Liaoning Institute of Sandy Land Control and Utilization, Liaoning Province, China (42°32′–42°51′ N, 121°53′–122°35′ E; average elevation, 226 m), which is located in the southeastern region of the Horqin Sandy Land, China. This region has a semiarid climate, with a mean annual precipitation of 474 mm, largely during June–August, and mean annual potential evaporation of approximately 1,580 mm (Song et al., 2020). The mean annual temperature is approximately 6.8°C, with minimum and maximum mean temperatures of −29.5 in January and 37.2°C in July, respectively. The zonal soil in this region is classified in the Semiaripsamment taxonomic group, which develops from sandy parent material through wind; the distributions of soil salinity, texture, and structures were homogeneous (Zhu et al., 2008). The main vegetation type is psammophytes, which are typical Inner Mongolia flora. The Zhanggutai Experimental Base was established in 1978; it covers an area of 2,620 hm^2^ and is characterized by flat, stable sand dunes and large *Pinus sylvestris* var. *mongolica* and *P. alba* plantations, interspersed with small patches of degraded grassland and *U. pumila* and *Pinus tabulaeformis* plantations.

### Experimental Design

**FIGURE 1:**
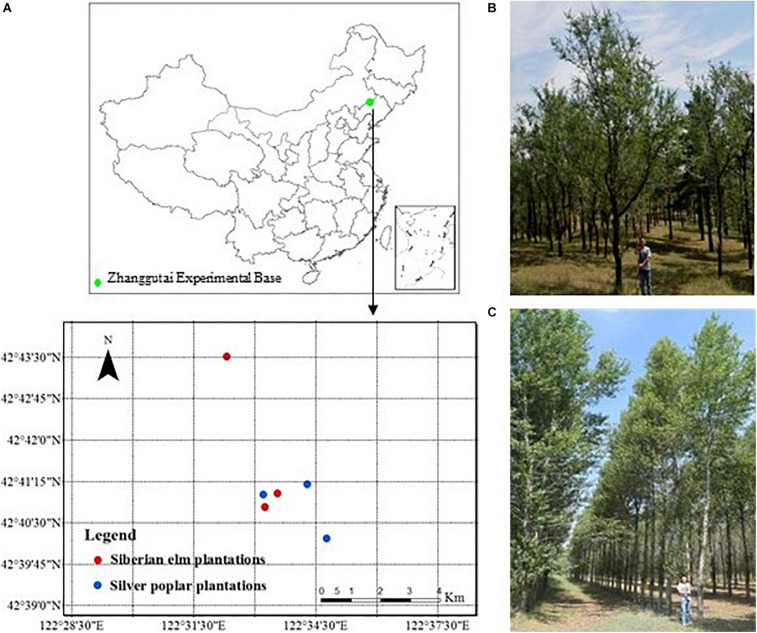
Location of the study area **(A)** and photos of the Siberian elm **(B)** and Silver poplar **(C)** plantations.

In 2017, three plots were selected among pure elm and poplar plantations, respectively on the Zhanggutai Experimental Base (Figure 1). These plots had similar site conditions and history and land management prior to afforestation. The plantations were established from non-vegetated sandy lands, and previous studies at the study region have shown that soil properties were homogeneous and similar in the non-vegetated sandy lands (Jiao, 1989; Zeng et al., 2009; Zhao et al., 2010). Based on investigation in the study region prior to afforestation, the distributions of soil nutrients were homogeneous and the C, N, and P concentrations were 3.15, 0.24, and 0.09 g kg^–1^, respectively. Thus, we considered that the initial soil properties were similar prior to afforestation, and the differences in soil C, N, and P stoichiometry after afforestation were induced by the different tree species plantations. All the selected plantations were planted on flat topography, and shared the same soil type (arenosols) and similar elevation (Table 1). All plots were within 10 km to ensure similar soil type and climatic condition. The selected trees were approximately 20 years old and therefore suitably represented the effects of plant and soil interactions on nutrients following afforestation (Zhao et al., 2010). No management techniques such as fertilization, pruning, or thinning were conducted in any of the plots. Three replicate subplots (20 m × 20 m) were established within each plot.

**TABLE 1:** The basic information of Siberian elm and Silver poplar plantations.

Forest species	Plot number	Longitude	Latitude	Elevation (m)	Stand age (year)	Density (trees ha^–1^)	Mean height (m)	Mean DBH (cm)	pH	Soil water content (%)	Soil particle-size distribution (%)		
											Clay (0–2 μm)	Silt (2–50 μm)	Sand (50–2,000 μm)
Siberian elm	1	122°33′15″ E	42°40′47″ N	218	**22**	780	5.4 ± 0.3	10.1 ± 0.4	7.3 ± 0.1	7.68 ± 0.09	3.03 ± 0.21	11.31 ± 2.06	85.66 ± 2.24
	2	122°32′19″ E	42°43′31″ N	238	23	800	5.6 ± 0.3	9.5 ± 0.9	7.3 ± 0.1	7.79 ± 0.66	2.94 ± 0.15	13.52 ± 1.49	83.54 ± 1.34
	3	122°33′34″ E	42°41′2″ N	214	25	910	6.5 ± 0.4	10.3 ± 0.4	7.4 ± 0.1	8.63 ± 0.1 8	3.21 ± 0.16	14.71 ± 2.16	82.08 ± 2.00
Silver poplar	1	122°34′17″ E	42°41′12″N	217	21	833	11.8 ± 0.6	14.2 ± 0.4	6.7 ± 0.1	6.43 ± 0.19	1.16 ± 0.08	5.08 ± 1.41	93.76 ± 1.49
	2	122°33′13″ E	42°41′1″ N	212.	23	825	13.2 ± 0.4	15.2 ± 0.8	6.7 ± 0.1	6.50 ± 0.26	1.56 ± 0.15	6.65 ± 1.06	91.79 ± 1.20
	3	122°34′46″ E	42°40′13″ N	206	24	920	12.0 ± 0.4	15.3 ± 0.7	6.7 ± 0.1	6.81 ± 0.07	1.82 ± 0.20	8.50 ± 1.13	89.68 ± 1.08

*DBH: diameter at breast height.*

Within each subplot, three healthy individuals with average diameter at breast height were randomly selected for plant sample collection. Leaf, branch, root, and soil samples were collected in mid-May, July, and September (i.e., spring, summer, and autumn). From each selected tree, we collected three branches from the upper, middle, and lower parts of the crown, and we sampled branches of similar diameter (approximately 5 mm) for each tree species during the different seasons. We selected mature leaves without diseases and/or insect pests as leaf samples. The fine roots (<2 mm) of each selected tree were excavated from several locations below the canopy by carefully removing the surrounding soil. Soil samples were simultaneously collected using a soil auger (diameter, 5 cm) at depths of 0–20, 20–40, and 40–60 cm. After removing the understory plants and surface litter, we randomly collected four soil samples within 1 m of the base of each selected tree; these were pooled into a single composite soil sample per tree. In mid-October, we collected newly fallen and undecomposed leaf litter from the litter layer under the canopy of each selected tree. Three replicate samples of leaves, branches, roots, litter, and soil were collected in each subplot. All plant samples were ground using a mechanical grinder after oven drying for 72 h at 60°C, and soil samples were air-dried after the removal roots and stones. All plant and soil samples were passed through a 0.25 mm sieve and then used to measure C, N, and P concentrations.

### Chemical Measurements

C concentrations in plant and soil samples were measured using the oil bath K_2_Cr_2_O_7_ titration method. To measure N and P concentrations, plant and soil samples were initially digested with H_2_SO_4_-H_2_O_2_ and H_2_SO_4_-HClO_4_, respectively, and then the total N and P concentrations were determined following the semi-micro Kjeldahl method using a Kjeldahl auto-analyzer (JY-SPD60, Beijing, China) and the colorimetric method using a spectrophotometer (T6, Beijing, China). Soil pH was measured using a soil/water ratio of 1:2.5 suspension. Soil water content determined from mass loss after drying for 12 h at 105°C (Bao, 2000). Soil particle size was analyzed using a Horiba Master Sizer (LA-300, Japan). Plant and soil C, N, and P concentrations were expressed as in dry mass (g kg^–1^), and C:N, C:P, and N:P ratios were calculated as mass ratios.

### Calculations

NuRE was calculated as follows:

(1) NuRE=N-mNl×MLCFNm×100%

where N_*m*_ and N_*l*_ are the nutrient concentrations in mature leaves (July) and litter leaves (October), respectively (Yan et al., 2016), and the MLCF is mass loss correction factor, specifically the dry mass ratio of litter leaves and mature leaves (van Heerwaarden et al., 2003).

Using the nutrient stoichiometry of plant organs and soils, the homeostatic regulation coefficient (*H*) was derived from the following model (Sterner and Elser, 2002):

(2) $$y=c+\frac{1}{H}\times \log x$$

where *y* is the N or P concentration or N:P for leaves, branches, and roots; *x* is the corresponding value in the soil layer; and *c* is a constant. If the regression relationship is not significant (*P* > 0.05), then 1/*H* is set at zero, and the organism is considered strictly homeostatic. If the regression relationship is significant (*P* < 0.05), then species with | 1/*H*| ≥ 1 are considered not to be homeostatic, where those with 0 < | 1/*H*| < 1 are classified as follows: 0 < | 1/*H*| < 0.25, homeostatic; 0.25 < | 1/*H*| < 0.5, weakly homeostatic; 0.5 < | 1/*H*| < 0.75, weakly plastic; or | 1/*H*| > 0.75, plastic (Persson et al., 2010; Bai et al., 2019).

### Statistical Analyses

For all datasets, Kolmogorov–Smirnov and Levene’s tests were conducted to test the normality and homogeneity of variances before statistical analysis. Two-way ANOVAs were used to test the effects of tree species and sampling time on C, N, and P concentrations and C:N, C:P, and N:P for each soil layer and tree organ. Duncan’s test was conducted for *post hoc* multiple comparisons. Nutrient stoichiometry of soil, plant, and leaf litter, as well as the NuRE, were compared between elm and poplar using the two-sample *t*-tests. All figures were prepared using SigmaPlot 10.0 software, and all data were analyzed using SPSS 16.0 software for Windows (SPSS Inc., Chicago, IL, United States). Significance was evaluated at a level of 0.05.

## Results

### Soil C, N, and P Stoichiometry

**FIGURE 2:**
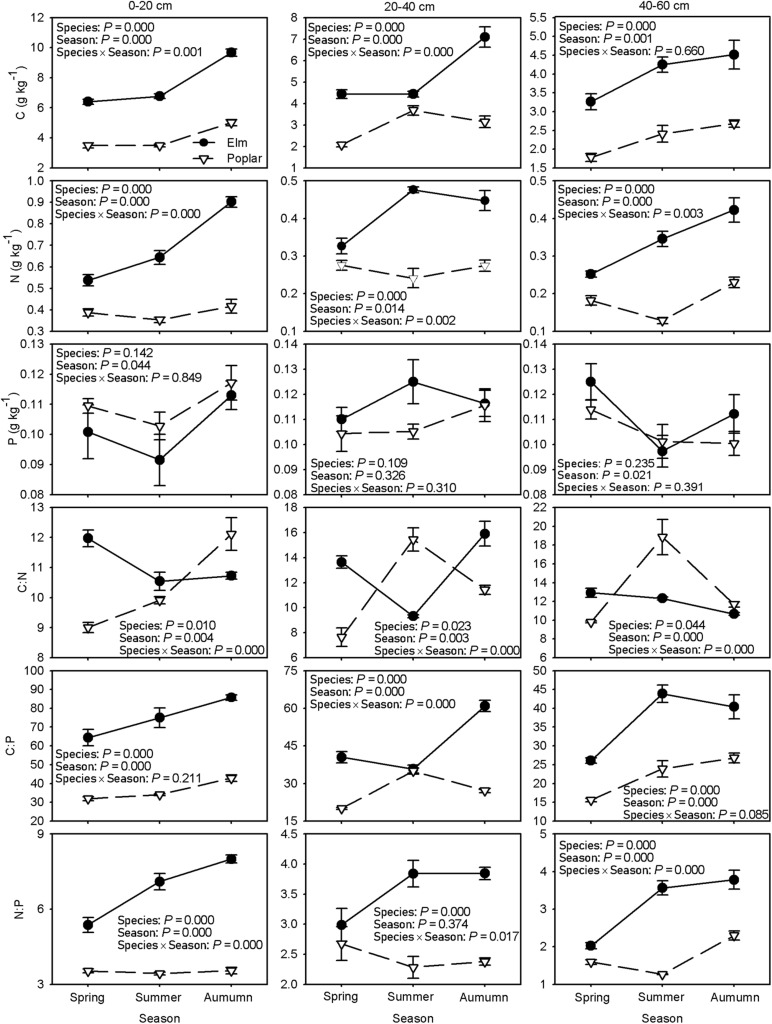
Differences in soil C, N, and P concentrations and C:N, C:P, and N:P across the growing season between Siberian elm and Silver poplar plantations (*n* = 3).

Soil C concentrations showed an upward trend during the growing season and were higher in the elm plantation than in the poplar plantation (Figure 2). Soil N concentrations tended to increase in the elm plantation but remained unchanged in the 0–20 and 20–40 cm soil layers in the poplar plantation over time; soil N concentrations were higher in the elm plantation than in the poplar plantation (Figure 2). Soil P concentrations tended to decrease and then increase in the 0–20 and 40–60 cm soil layers but remained unchanged in the 20–40 cm soil layer in both the elm and poplar plantations over time, and no significant differences were found between plantations (Figure 2).

Soil C:N ratio in the 0–20 and 40–60 cm soil layers showed a downward trend but decreased and then increased in the 20–40 cm soil layer in the elm plantation over time, while it showed an upward trend in the 0–20 cm soil layer but initially decreased and then increased in the 20–40 and 40–60 cm soil layers in the poplar plantation over time. Higher soil C:N in the elm plantation than in the poplar plantation was found in spring (Figure 2). Generally, soil C:P showed an upward trend over time and was greater in the elm plantation than in the poplar plantation (Figure 2). Soil N:P tended to increase in the elm plantation but remained stable in the 0–20 and 20–40 cm soil layers in the poplar plantation during the growing season; soil N:P was higher in the elm plantation than in the poplar plantation (Figure 2).

### Plant C, N, and P Stoichiometry and Nutrient Resorption

**FIGURE 3:**
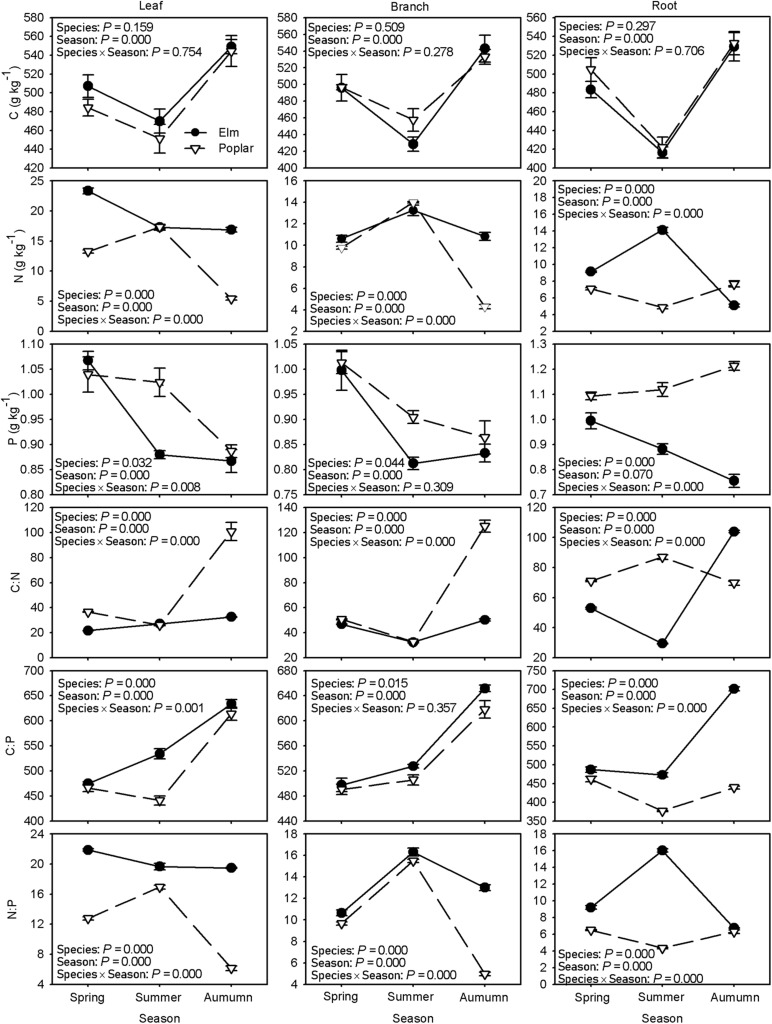
Differences in organ C, N, and P concentrations and C:N, C:P, and N:P across the growing season between Siberian elm and Silver poplar plantations (*n* = 3).

Tree species did not have a significant effect on C concentrations in all three organs, although sampling time had a significant effect, with an initial decreasing and subsequent increasing trend (Figure 3). N concentrations decreased in leaves but increased and then decreased in branches and roots among elm samples during the growing season, whereas they increased and then decreased in poplar leaves and branches but showed the opposite trend in poplar roots. N concentrations in leaves during spring and autumn, branches during autumn, and roots during spring and summer were higher in elm than in poplar (Figure 3). P concentrations tended to decrease in the leaves, branches, and roots of elm and in the leaves and branches of poplar, whereas they increased in poplar roots during the growing season. Leaf and branch P concentrations during summer and root P concentrations were higher in poplar than in elm (Figure 3).

In elm samples, C:N ratio increased in leaves, whereas in branches and roots, it initially decreased and then increased during the growing season; in poplar samples, C:N ratio initially decreased and then increased in leaves and branches but showed the opposite trend in roots. Leaf and branch C:N ratio were lower whereas root C:N ratio were higher in elm than in poplar during autumn (Figure 3). C:P showed an upward trend in all three organs of both elm and poplar samples over time, with the exception of poplar roots, in which it decreased and then increased. Leaf C:P during summer and root C:P during summer and autumn were higher in elm than in poplar (Figure 3). N:P decreased in leaves but initially increased and then decreased in branches and roots in elm samples over time, while it increased and then decreased in leaves but showed the opposite trend in roots in poplar samples over time. Tree species had a significant effect on N:P, with higher in elm than in poplar except for branches during spring and summer and roots during autumn (Figure 3).

**FIGURE 4:**
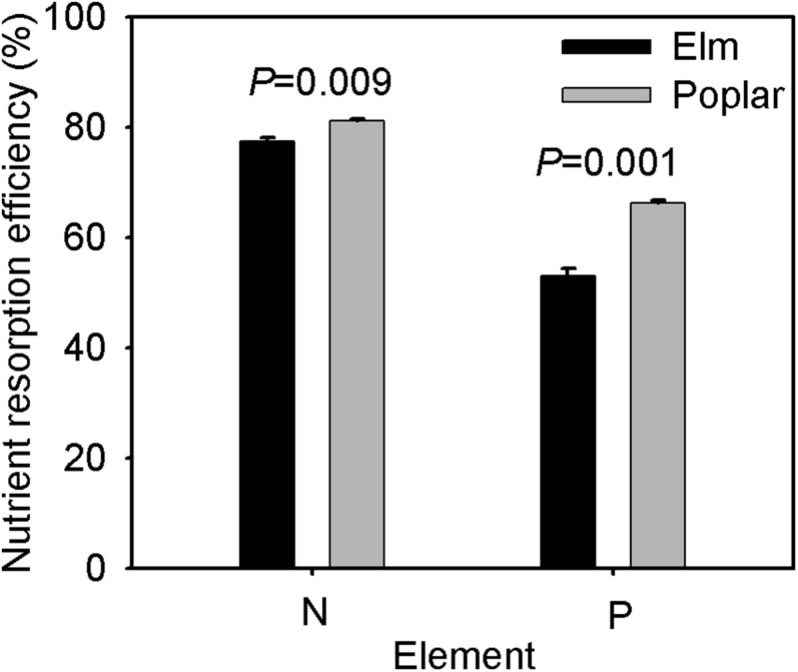
N and P resorption efficiencies of Siberian elm and Silver poplar (*n* = 3).

Leaf litter N and P concentrations were significantly higher, but C:N and C:P were lower, in the elm plantation than in the poplar plantation, and no significant differences were found in C concentrations or N:P between the two plantations (Table 2). N and P resorption efficiencies were significantly greater in the poplar plantation than in the elm plantation (Figure 4).

**TABLE 2:** The C, N, and P concentrations and their ratios in leaf litter of elm and poplar plantations.

Tree species	C (g kg^–1^)	N (g kg^–1^)	P (g kg^–1^)	C:N	C:P	N:P
Siberian elm	449.54 ± 10.48	4.79 ± 0.20*	0.51 ± 0.02*	93.94 ± 1.83*	883.35 ± 14.56*	9.40 ± 0.03
Silver poplar	419.39 ± 13.56	3.84 ± 0.16	0.41 ± 0.01	109.30 ± 1.23	1,034.27 ± 8.93	9.47 ± 0.16

*Indicate significant differences between the tree species at P < 0.05.

### Stoichiometric Homeostasis

**FIGURE 5:**
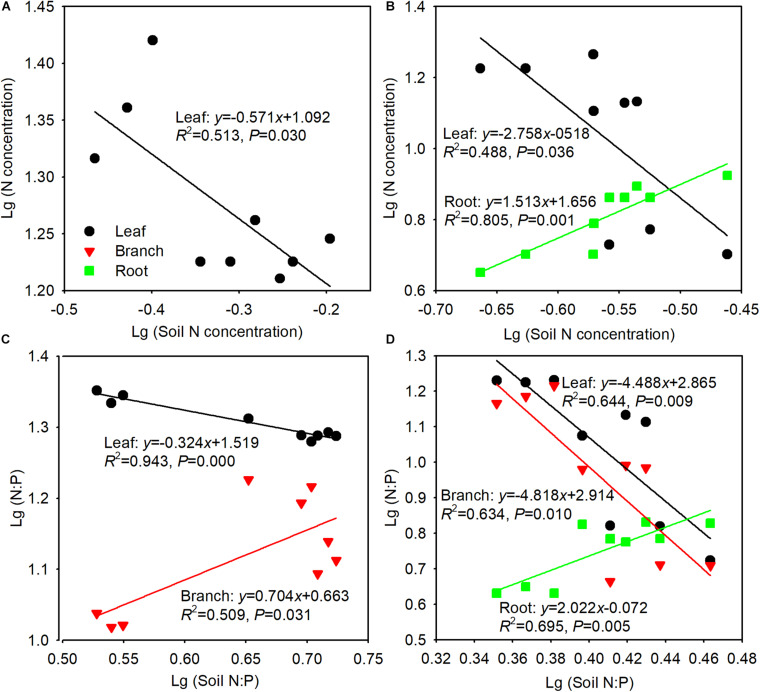
Relationships between tree organ and soil N concentration in Siberian elm **(A)** and Silver poplar **(B)** plantations, and N:P in Siberian elm **(C)** and Silver poplar **(D)** plantations. Only the cases with significance are shown. (*n* = 9 with 3 plots × 3 sampling seasons).

We detected the degree of stoichiometric homeostasis of N, P, and N:P in leaves, branches, and roots of elm and poplar, only significant relationships were shown in Figure 5. We found strict N concentration homeostasis in elm branches and roots (*P* > 0.05) and weak plasticity in elm leaves. However, N concentrations were not homeostatic in poplar leaves or roots and were strictly homeostatic in poplar branches (*P* > 0.05) (Table 3). No significant relationships were found in P concentrations between soil and organs (*P* > 0.05), thus P concentrations were strictly homeostatic in the leaves, branches, and roots of both tree species (Table 3). N:P was weakly homeostatic, weakly plastic, and strictly homeostatic in elm leaves, branches, and roots, respectively, whereas N:P was not homeostatic in poplar leaves, branches, or roots, decreasing in leaves and branches and increasing in roots as soil N:P increased (Table 3).

**TABLE 3:** Regression slopes (1/*H*) of relationships between plant and soil nutrient stoichiometry of Siberian elm and Silver poplar.

	Siberian elm			Silver poplar		
	Leaf	Branch	Root	Leaf	Branch	Root
N	–0.571	0	0	–2.758	0	1.513
P	0	0	0	0	0	0
N:P	–0.324	0.704	0	–4.488	–4.818	2.022

*If the regression relationship is not significant (P > 0.05), then 1/H is set at zero, and the organism is considered strictly homeostatic. If the regression relationship is significant (P < 0.05), then species with | 1/H| ≥ 1 are considered not to be homeostatic, where those with 0 < | 1/H| < 1 are classified as follows: 0 < | 1/H| < 0.25, homeostatic; 0.25 < | 1/H| < 0.5, weakly homeostatic; 0.5 < | 1/H| < 0.75, weakly plastic; or | 1/H| > 0.75, plastic.*

## Discussion

### Comparison of Soil C, N, and P Stoichiometry Between Elm and Poplar Plantations

Afforestation can improve plant and soil nutrient concentrations and stocks, soil quality, and vegetation structure via more efficient use of resources for primary production (Nosetto et al., 2006). Afforestation increases water-holding capacity and nutrient retention (Evrendilek et al., 2004), increasing the efficacy of C sequestration and enhancing ecosystem biodiversity and resilience in semiarid regions (Hernandez-Ramirez et al., 2011). In this study, compared with nearby wild grassland without afforestation (the C, N and P concentrations were 4.50, 0.31 and 0.11 g kg^−1^ at 0–20 cm; 2.35, 0.12 and 0.10 g kg^−1^ at 20–40 cm; and 1.54, 0.10 and 0.10 g kg^−1^ at 40–60 cm, respectively), both elm and poplar plantations increased the concentrations of soil C and N, especially in deeper soil layers, whereas there was no significant influence on P concentrations (Figure 2). Similar results were reported for *P. sylvestris* var. *mongolica* afforestation in the Horqin Sandy Land (Li et al., 2012); however, Zhao et al. (2008) reported that afforestation significantly reduced soil P concentrations but had no significant effect on soil C or N concentrations, perhaps due to differences in tree species or stand density. Thus, the choice of afforestation species and plantation management technique may be key factors for successful afforestation (Zhao et al., 2008; Bai et al., 2019; Ahmed et al., 2020).

As predicted, the soil C and N concentrations were greater in the elm plantation than in the poplar plantation (Figure 2), perhaps due to possible nutrient consumption for plant growth and nutrient return in the form of leaf litter. Poplar is a fast-growing, high-yield tree species (Ahmed et al., 2020) and had much larger total biomass than elm (Table 1), while it had similar N concentrations and higher P concentrations in leaves and branches compared with elm during summer. These findings indicated more nutrients were absorbed, assimilated, and sequestrated by poplar, which may contribute to lower soil C and N concentrations in the poplar plantation than in the elm plantation. Additionally, our elm plantation samples had higher N and P concentrations and lower C:N and C:P in leaf litter (Table 2) and lower N and P resorption efficiencies in leaves (Figure 4) compared with poplar samples, indicating the return of high-quality litter to soil, which accelerated litter decomposition and nutrient mineralization in the elm plantation. Afforestation not only affects soil nutrients but also affects soil texture. Soil granulometric composition significantly correlated with soil nutrient concentrations on the sandy land after afforestation (Deng et al., 2017), thus, more soil clay and silt contents (Table 1) maybe one of the reasons for higher soil C and N concentrations in the elm plantation (Deng et al., 2017). However, there was no significant difference in soil P concentrations between the poplar and elm plantations (Figure 2), which was inconsistent with predictions, perhaps due to the differences in soil P sources and transformation processes compared with soil C and N. The accumulation of soil C and N is driven mainly by the decomposition of plant litter and dead roots, whereas soil P transformation is driven primarily by phosphate decomposition which requires long periods of time (Deng et al., 2019). The N and P cycles can become decoupled under drought stress (Delgado-Baquerizo et al., 2013), and soil P diffusivity is more sensitive to soil water than that of N (Lambers et al., 2008); therefore, cation exchange and P sorption capacity are very low in sandy soil (Leinweber et al., 1999). Thus, the total P in the soil remained at levels too low for adequate absorption and use by plants (Ma et al., 2009). A similar result was reported for *Pinus radiata* in a temperate Andisol soil, in which the P concentrations remained unchanged following afforestation (Farley and Kelly, 2004).

Generally, the soil C concentrations showed an upward trend in both plantations over time, and the increase was greater in the elm plantation than in the poplar plantation (Figure 2). Hu et al. (2016) reported that soil C sequestration was driven mainly by root input rather than leaf litter input after afforestation. Higher fine root biomass was found in the elm plantation than in the poplar plantation (Wang et al., 2014), which produced more root litter and exudates to facilitate soil C transformation processes. Soil N concentrations showed an increase trend in the elm plantation, but no significant changes were observed in the 0–20 or 20–40 cm soil layers in the poplar plantation during the growing season (Figure 2), perhaps due to the leaf litter decomposition rate (Wojciech et al., 2019). Elm leaves are small and soft, whereas those of poplar are larger and tougher with a thicker wax layer. Elm had higher litter decomposition and nutrient release rates than does poplar (Zhang et al., 2013). Soil P concentrations tended to decrease and then increase in the 0–20 cm soil layer in both plantations (Figure 2), perhaps because more P in topsoil was absorbed by plants during summer. P cycling is driven mainly by plant P demand and sustained by forest leaf litter inputs (Chen et al., 2008).

Soil C:N:P stoichiometry is an important indicator of nutrient cycling and elemental limitations in plants (Luo et al., 2020). In our study, the average surface soil C:N, C:P, and N:P of the two plantations were 10.7, 55.6, and 5.1, respectively (Figure 2), which were lower than average values in China (14.4, 136.0, and 9.3) and worldwide (14.3, 186.0, and 13.0) (McGroddy et al., 2004; Tian et al., 2010). Compared with sandy grassland (Liu et al., 2013) and shrubland (Yang and Liu, 2019), we found lower C:N and higher C:P and N:P in the plantations, implying that soil P content is lower in forest plantations in the Horqin Sandy Land. This result may have been caused by greater sensitivity of P than N ion movement to soil moisture conditions (Walbridge, 2000; Smith, 2002; He and Dijkstra, 2014), leading to a greater dependence of soil P availability on soil water availability under the drought conditions at the study site (Yang and Liu, 2019). Furthermore, most of the P absorbed and assimilated by trees is sequestrated within biomass (Kuznetsova et al., 2011; Yan et al., 2017). Soil C:P has negative effects on soil P availability (Li et al., 2016). In our study, soil C:P and N:P were generally greater in the elm plantation than in the poplar plantation (Figure 2) indicating lower soil P availability in the elm plantation relative to the poplar plantation.

### Comparison of Plant C, N, and P Stoichiometry Between Elm and Poplar Plantations

C, N, and P concentrations and stoichiometric ratios in different plant organs can reflect adaptive strategies to various regimes in terms of nutrient uptake, allocation, and utilization during plant growth (Niklas and Cobb, 2005). C concentrations did not differ significantly among plantation species, whereas they decreased and then increased over time (Figure 3). Lower C concentrations often lead to higher specific leaf area and photosynthetic and growth rates (Niklas and Cobb, 2005), implying faster growth for both tree species during summer than during spring and autumn. Inconsistent with our second prediction, poplar had higher P concentrations and lower C:P than elm during summer, implying faster growth rate and more biomass accumulation. However, P concentrations and C:P were similar in poplar leaf and branch compared with elm during spring and autumn (Figure 3). Drought stress can induce a decrease in available soil P (He and Dijkstra, 2014), thus limited P absorption from soil leads to low P concentrations in plants to maintain C assimilation in arid and nutrient-poor environments (He and Dijkstra, 2014), which may explain the similar P concentrations observed in the leaves and branches between poplar and elm during spring and autumn. Drought often induces xylem embolism in taller tree species (McDowell et al., 2008), and water transport failure can affect nutrient translocation and allocation (He and Dijkstra, 2014). Poplar is taller and more susceptible to hydraulic failure in sandy regions (Song et al., 2021), which may lead to nutrient accumulation in roots. This process may also explain the higher NuRE of poplar than elm (Figure 4). Inconsistent with our second prediction, N concentrations and C:N in leaves and branches were similar between elm and poplar during summer, although lower N concentrations and higher C:N in leaves and branches were found in poplar than in elm during autumn (Figure 3). These findings may because of growth dilution effects after summer caused by the greater biomass and growth rate of poplar compared with those of elm. In poplar, N concentrations were higher in leaves and branches but lower in roots during summer than those during spring and autumn (Figure 3), implying that more resources are allocated to leaves and branches during the rapid growth season to promote the growth of aerial plant part. N and P concentrations increased in roots but decreased in leaves and branches during autumn (Figure 3), which indicated that most N and P were reabsorbed and transferred to roots for storage, implying a more conservative nutrient use strategy that benefits sprouting and new leaf growth during the following spring.

Elm organ N and P concentrations decreased in autumn (Figure 3), whereas N and P resorption efficiencies were lower in elm than in poplar (Figure 4). This finding indicates the return of high-quality leaf litter to the soil, leading to greater N and P acquisition by elm via root uptake and implying more efficient nutrient cycles in the plant–litter–soil system. The N concentrations in branches and roots were higher during summer than during spring and autumn, with no significant differences between these organs (Figure 3). This implies that resource allocation between aerial and underground plant parts may be more balanced in elm than in poplar, promoting root growth during the rapid growing season for greater absorption of nutrient and water.

The leaf N:P can be used to determine potential N or P limitations for plant growth, and a ratio < 14 indicates N limitation, whereas a ratio > 16 indicates P limitation (Koerselman and Meuleman, 1996). In this study, the leaf N:P of elm was generally > 16 during the entire growing season (Figure 3), indicating P limitation for elm growth. However, the leaf N:P of poplar was > 16 in summer but < 14 in spring and autumn (Figure 3), which indicates that poplar experienced more P limitation during the fast-growing season and more N limitation during the early and late growing seasons. Generally, elm may be more susceptible to P limitation as it had higher N:P than poplar.

### Comparison of Stoichiometric Homeostasis Between Elm and Poplar Plantations

Stoichiometric homeostasis reflects the balance between resource consumption and storage in plants during growth period (Blouin et al., 2012), and it is positively correlated with vegetation stability (Yu et al., 2010). Stoichiometric homeostasis has been used to study the mechanisms of plant adaption to environmental change (Wang et al., 2019), and it has been compared among tree species with similar age following afforestation on the Loess Plateau of China (Bai et al., 2019) and in arid mining subsidence areas (Xiao et al., 2021). Although the soil granulometric composition may influence nutrient concentration in tissue, it would not affect homeostatic analysis. Variations in plant nutrient concentrations are the integrated results of soil improvement following afforestation (Bai et al., 2019). The homeostasis analysis is based on the current N and P concentration and N:P in soils and plants (Bai et al., 2019), regardless of the soil granulometric composition changes. In this study, both elm and poplar exhibited N and P concentration and N:P ratio homeostasis to some extent across the growing season (Figure 5), indicating relatively conservative nutrient use in both species, which improves their adaptation to this arid and nutrient-deficient environment. The maintenance of stable elemental composition in the plant body in a changeable environment is beneficial for growth, development and survival (Blouin et al., 2012). To minimize the effects of tree size, we only compared N:P homeostasis between elm and poplar. Consistent with our third hypothesis, elm generally showed greater N:P homeostasis than did poplar (Figure 5), indicating that elm may have more developed nutrient modulation systems than poplar, or that elm contains more functional materials, leading to a faster response to nutrient regime changes (Bai et al., 2019). Native species such as elm have a longer life history in a given local environment, which could allow it to adapt better to adverse environmental conditions, thereby improving ecosystem stability in the elm plantation compared with the poplar plantation. However, stoichiometric homeostasis is coupled to tree growth and development (Wang et al., 2019). In future studies, the stoichiometric homeostasis is needed to be evaluated in the two tree species of different age and size to better understand the mechanisms of nutrient conservation.

Limiting elements in plants with homeostasis generally have low variability and environmental sensitivity (Han et al., 2011); thus, they are the main regulators of homeostasis (Sterner and Elser, 2002). Leaves, branches, and roots in both elm and poplar trees were found to have strict P homeostasis (Table 3), indicating that P may be the main nutrient limiting factor for the growth of mature elm and poplar plantations. Similar results were found in *P. sylvestris* var. *mongolica* plantations (Zhao et al., 2009) and *Caragana* shrubs (Yang and Liu, 2019) in the same region.

The degree of stoichiometric homeostasis appears to vary among organs (Bai et al., 2019), reflecting a fundamental trade-off in nutrient investment and allocation among organs (Gu et al., 2017). In this study, elm branch and root N concentrations and root N:P showed strict homeostasis, whereas the leaf N concentrations and N:P were weakly plastic and weakly homeostatic, respectively (Table 3). These results are inconsistent with those of previous studies demonstrating that leaf homeostasis is often greater than that of other organs such as branches, roots, and fruits (Bai et al., 2019; Wang et al., 2019), perhaps because leaf nutrient contents are constrained within a certain range to provide optimal physiological traits for the maintenance of survival and growth (Aerts and Chapin, 2000). Elm can survive after disastrous weather, insect or disease events, even if all leaves are lost. Therefore, maintaining the nutrient balance in elm roots may be an adaptive strategy in arid and barren environments. In poplar, the N concentrations and N:P among the three organs were not homeostatic, except for the N concentrations in branches (Table 3); the N concentrations and N:P decreased in leaves and branches but increased in roots as soil N concentrations and N:P increased (Figures 5B,D). These findings indicate that poplar coordinates nutrient allocation among organs and nutrient translocation between aerial (leaf and branch) and underground (root) part, which showed opposite trends. When poplar experienced nutrient limitation, it decreased nutrient supply to the aerial parts and increased nutrient storage in underground parts. Poplar produces many root shoots and can sprout from roots in the spring following nutrient limitation, even if the aerial parts have died.

## Conclusion

In this study, nutrient conservation, use mechanisms, and stoichiometric homeostasis traits differed between elm and poplar plantations in the Horqin Sandy Land of China. Elm had lower organ N and P concentrations in autumn but greater litter N and P, and soil C and N concentrations, which enhanced nutrient cycling in the plant–litter–soil system. Elm evenly allocated N and P contents between aerial and underground parts. In contrast, poplar had higher root N and P concentrations in autumn and higher N and P resorption efficiencies but lower soil C and N concentrations, implying a more conservative nutrient use strategy and more developed internal nutrient cycles. Poplar had higher P concentrations and lower C:P than elm and allocated more N and P to leaves and branches during summer, implying faster growth rate and greater biomass, which contributed to lower soil nutrient concentrations. These traits are beneficial for early poplar growth, although stand degradation is expected to occur once soil nutrients can no longer sustain the nutritive requirements for growth. Generally, elm exhibited greater N:P homeostasis than poplar. Elm showed greater homeostasis in roots than in leaves and branches, whereas poplar coordinated nutrient allocation among organs. P was the main nutrient limiting factor in both elm and poplar plantations. Overall, elm was more adaptable to the arid, nutrient-deficient environment in terms of fostering soil nutrient accumulation and improving nutrient cycles in plant–litter–soil systems of the Horqin Sandy Land.

## Data Availability Statement

The raw data supporting the conclusions of this article will be made available by the authors, without undue reservation.

## Author Contributions

KW and RZ conceived and designed the study. KW and EN performed the experiments. KW and TY wrote the manuscript. TY and LS reviewed and edited the manuscript. All authors read and approved the manuscript.

## Conflict of Interest

The authors declare that the research was conducted in the absence of any commercial or financial relationships that could be construed as a potential conflict of interest.

## References

[B1] Aerts R. Chapin F. I. (2000). The mineral nutrition of wild plants revisited: a re-evaluation of processes and patterns. *Adv. Ecol. Res.* 30 1–67. 10.1016/S0065-2504(08)60016-1

[B2] Ahmed A. K. M. Fu Z. X. Ding C. J. Jiang L. P. Han X. D. Yang A. G. (2020). Growth and wood properties of a 38-year-old *Populus simonii* × *P. nigra* plantation established with different densities in semi-arid areas of northeastern China. *J. For. Res.* 31 497–506. 10.1007/s11676-019-00887-z

[B3] Bai X. J. Wang B. R. An S. S. Zeng Q. C. Zhang H. X. (2019). Response of forest species to C:N:P in the plant-litter-soil system and stoichiometric homeostasis of plant tissues during afforestation on the Loess Plateau, China. *Catena* 183:104186. 10.1016/j.catena.2019.104186

[B4] Bao S. D. (2000). *Soil and Agriculture Chemistry Analysis.* Beijing: China Agriculture Press.

[B5] Blouin M. Mathieu J. Leadley P. W. (2012). Plant homeostasis, growth and development in natural and artificial soils. *Ecol. Complex.* 9 10–15. 10.1016/j.ecocom.2011.11.001

[B6] Bui E. N. Henderson B. L. (2013). C:N:P stoichiometry in Australian soils with respect to vegetation and environmental factors. *Plant Soil* 373 553–568. 10.1007/s11104-013-1823-9

[B7] Capozzi F. Palma A. D. Paola F. D. Giugni M. Iavazzo P. Topa M. E. (2018). Assessing desertification in sub-Saharan peri-urban areas: case study applications in Burkina Faso and Senegal. *J. Geochem. Explor.* 190 281–291. 10.1016/j.gexplo.2018.03.012

[B8] Chen C. R. Condron L. M. Xu Z. H. (2008). Impacts of grassland afforestation with coniferous trees on soil phosphorus dynamics and associated microbial processes: a review. *For. Ecol. Manag.* 255 396–409. 10.1016/j.foreco.2007.10.040

[B9] Chu X. Zhan J. Y. Li Z. H. Zhang F. Qi W. (2019). Assessment on forest carbon sequestration in the Three-North Shelterbelt Program region, China. *J. Clean. Prod.* 215 382–389. 10.1016/j.jclepro.2018.12.296

[B10] Daufresne T. Loreau M. (2001). Ecological stoichiometry, primary producer-decomposer interactions, and ecosystem persistence. *Ecology* 82 3069–3082.

[B11] Delgado-Baquerizo M. Maestre F. T. Gallardol A. Bowker M. A. Wallenstein M. D. Quero J. L. (2013). Decoupling of soil nutrient cycles as a function of aridity in global drylands. *Nature* 502 672–676. 10.1038/nature12670 24172979

[B12] Deng J. Wang S. Ren C. J. Zhang W. Zhao F. Z. Li X. F. (2019). Nitrogen and phosphorus resorption in relation to nutrition limitation along the chronosequence of Black Locust (*Robinia pseudoacacia* L.) plantation. *Forests* 10:261. 10.3390/f10030261

[B13] Deng J. F. Li J. H. Deng G. Zhu H. Y. Zhang R. H. (2017). Fractal scaling of particle-size distribution and associations with soil properties of Mongolian pine plantations in the Mu Us Desert, China. *Sci. Rep.* 7:6742. 10.1038/s41598-017-06709-8 28751742 PMC5532370

[B14] Elser J. Fagan W. Kerkhoff A. Swenson N. Enquist B. (2010). Biological stoichiometry of plant production: metabolism, scaling and ecological response to global change. *New Phytol.* 186 593–608. 10.1111/j.1469-8137.2010.03214.x 20298486

[B15] Evrendilek F. Celik I. Kilic S. (2004). Changes in soil organic carbon and other physical soil properties along adjacent Mediterranean forest, grassland, and cropland ecosystems in Turkey. *J. Arid Environ.* 59 743–752. 10.1016/j.jaridenv.2004.03.002

[B16] Fang Z. Li D. D. Jiao F. Yao J. Du H. T. (2019). The latitudinal patterns of leaf and soil C:N:P stoichiometry in the Loess Plateau of China. *Front. Plant Sci.* 10:85. 10.3389/fpls.2019.00085 30949183 PMC6436477

[B17] Farley K. A. Kelly E. F. (2004). Effects of afforestation of a páramo grassland on soil nutrient status. *For. Ecol. Manag.* 195 281–290. 10.1016/j.foreco.2003.12.015

[B18] Gu Q. Zamin T. J. Grogan P. (2017). Stoichiometric homeostasis: a test to predict tundra vascular plant species and community-level response to climate change. *Arctic Sci.* 3 320–333. 10.1139/as-2016-0032

[B19] Han W. X. Fang J. Y. Reich P. B. Woodward F. L. Wang Z. H. (2011). Biogeography and variability of eleven mineral elements in plant leaves across gradients of climate, soil and plant functional type in China. *Ecol. Lett.* 14 788–796. 10.1111/j.1461-0248.2011.01641.x 21692962

[B20] He M. Z. Dijkstra F. A. (2014). Drought effect on plant nitrogen and phosphorus: a meta-analysis. *New Phytol.* 204 924–931. 10.1111/nph.12952 25130263

[B21] Hernandez-Ramirez G. Sauer T. J. Cambardella C. A. Brandle J. R. James D. E. (2011). Carbon sources and dynamics in afforested and cultivated corn belt soils. *Soil Sci. Soc. Am. J.* 75 216–225. 10.2136/sssaj2010.0114

[B22] Hessen D. O. Ågren G. I. Anderson T. R. Elser J. J. Ruiter P. C. (2004). Carbon sequestration in ecosystems: the role of stoichiometry. *Ecology* 85 1179–1192. 10.1890/02-0251

[B23] Hu Y. L. Zeng D. H. Ma X. Q. Chang S. X. (2016). Root rather than leaf litter input drives soil carbon sequestration after afforestation on a marginal cropland. *For. Ecol. Manag.* 362 38–45. 10.1016/j.foreco.2015.11.048

[B24] Jiao S. R. (1989). *Structure and Function of Sandy Forest Ecosystems in Zhanggutai.* Liaoning: Scinece and Technology Press.

[B25] Kobe R. K. Lepczyk C. A. Iyer M. (2005). Resorption efficiency decreases with increasing green leaf nutrients in a global data set. *Ecology* 86 2780–2792. 10.1890/04-1830

[B26] Koerselman W. Meuleman A. F. M. (1996). The vegetation N:P ratio: a new tool to detect the nature of nutrient limitation. *J. Appl. Ecol.* 33 1441–1450. 10.2307/2404783

[B27] Kuznetsova T. Lukjanova A. Mandre M. Lõhmus K. (2011). Aboveground biomass and nutrient accumulation dynamics in young black alder, silver birch and Scots pine plantations on reclaimed oil shale mining areas in Estonia. *For. Ecol. Manag.* 262 56–64. 10.1016/j.foreco.2010.09.030

[B28] Lambers H. Chapin F. S. Pons T. L. (2008). *Plant Physiological Ecology.* New York, NY: Springer.

[B29] Leinweber P. Meissner R. Eckhardt K. U. Seeger J. (1999). Management effects on forms of phosphorus in soil and leaching losses. *Eur. J. Soil Sci.* 50 413–424. 10.1046/j.1365-2389.1999.00249.x

[B30] Li Y. Niu S. L. Yu G. R. (2016). Aggravated phosphorus limitation on biomass production under increasing nitrogen loading: a meta-analysis. *Glob. Chang. Biol.* 22 934–943. 10.1111/gcb.13125 26463578

[B31] Li Y. Q. Awada T. Zhou X. H. Shang W. Chen Y. P. Zuo X. A. (2012). Mongolian pine plantations enhance soil physico-chemical properties and carbon and nitrogen capacities in semi-arid degraded sandy land in China. *Appl. Soil Ecol.* 56 1–9. 10.1016/j.apsoil.2012.01.007

[B32] Li Y. Q. Zhao H. L. Zhao X. Y. Zhang T. H. Chen Y. P. (2006). Biomass energy, carbon and nitrogen stores in different habitats along a desertification gradient in the Semiarid Horqin Sandy Land. *Arid Land Res. Manag.* 20 43–60. 10.1080/15324980500369285

[B33] Lindroth R. L. Clair S. B. S. (2013). Adaptations of quaking aspen (*Populus tremuloides* Michx.) for defense against herbivores. *For. Ecol. Manag.* 299 14–21. 10.1016/j.foreco.2012.11.018

[B34] Liu M. Dries L. Heijman W. Huang J. Zhu X. Hu Y. (2018). The impact of ecological construction programmes on grassland conservation in Inner Mongolia, China. *Land Degrad. Dev.* 29 326–336. 10.1002/ldr.2692

[B35] Liu R. T. Zhao H. L. Zhao X. Y. Zhu F. (2013). Effects of cultivation and grazing exclusion on the soil macro-faunal community of semiarid sandy grasslands in northern China. *Arid Land Res. Manag.* 27 377–393. 10.1080/15324982.2013.787470

[B36] Luo X. Z. Hou E. Q. Chen J. Q. Li J. Zhang L. L. Zang X. W. (2020). Dynamics of carbon, nitrogen, and phosphorus stocks and stoichiometry resulting from conversion of primary broadleaf forest to plantation and secondary forest in subtropical China. *Catena* 193:104606. 10.1016/j.catena.2020.104606

[B37] Ma B. Zhou Z. Y. Zhang C. P. Zhang G. Hu Y. J. (2009). Inorganic phosphorus fractions in the rhizosphere of xerophytic shrubs in the Alxa Desert. *J. Arid Environ.* 73 55–61. 10.1016/j.jaridenv.2008.08.006

[B38] Manzoni S. Jackson R. B. Trofymow J. A. Porporato A. (2008). The global stoichiometry of litters nitrogen mineralization. *Science* 321 684–686. 10.1126/science.1159792 18669860

[B39] McDowell N. G. Pockman W. T. Allen C. D. Breshears D. D. Cobb N. Kolb T. (2008). Mechanisms of plant survival and mortality during drought: why do some plants survive while others succumb to drought? *New Phytol.* 178 719–739. 10.1111/j.1469-8137.2008.02436.x 18422905

[B40] McGroddy M. E. Daufresne T. Hedin L. O. (2004). Scaling of C:N:P stoichiometry in forests worldwide: implications of terrestrial Redfield-type ratios. *Ecology* 85 2390–2401.

[B41] Mulder C. Elser J. J. (2009). Soil acidity, ecological stoichiometry and allometric scaling in grassland food webs. *Glob. Chang. Biol.* 15 2730–2738. 10.1111/j.1365-2486.2009.01899.x

[B42] Niklas K. J. Cobb E. D. (2005). N, P, and C stoichiometry of *Eranthis hyemalis* (Ranunculaceae) and the allometry of plant growth. *Am. J. Bot.* 92 1256–1263. 10.3732/ajb.92.8.1256 21646146

[B43] Nosetto M. D. Jobbágy E. G. Paruelo J. M. (2006). Carbon sequestration in semi-arid rangelands: comparison of *Pinus ponderosa* plantations and grazing exclusion in NW Patagonia. *J. Arid Environ.* 67 142–156. 10.1016/j.jaridenv.2005.12.008

[B44] Peng H. Y. Chen Y. H. Yan Z. B. Han W. X. (2016). Stage-dependent stoichiometric homeostasis and responses of nutrient resorption in *Amaranthus mangostanus* to nitrogen and phosphorus addition. *Sci. Rep.* 6:37219. 10.1038/srep37219 27849041 PMC5110967

[B45] Persson J. Fink P. Goto A. James M. H. Jayne J. Satoshi K. (2010). To be or not to be what you eat: regulation of stoichiometric homeostasis among autotrophs and heterotrophs. *Oikos* 119 741–751. 10.1111/j.1600-0706.2009.18545.x

[B46] Sinsabaugh R. L. Lauber C. L. Weintraub M. N. Ahmed B. Allison S. D. Crenshaw C. (2008). Stoichiometry of soil enzyme activity at global scale. *Ecol. Lett.* 11 1252–1264. 10.1111/j.1461-0248.2008.01245.x 18823393

[B47] Smith F. W. (2002). The phosphate uptake mechanism. *Plant Soil* 245 105–114. 10.1023/A:1020660023284

[B48] Song L. N. Zhu J. J. Li M. C. Zhang J. X. Wang K. Lü L. Y. (2020). Comparison of water-use patterns for non-native and native woody species in a semiarid sandy region of Northeast China based on stable isotopes. *Environ. Exp. Bot.* 174:103923. 10.1016/j.envexpbot.2019.103923

[B49] Song L. N. Zhu J. J. Zhang T. Wang K. Wang G. C. Liu J. H. (2021). Higher canopy transpiration rates induced dieback in poplar (*Populus × xiaozhuanica*) plantations in a semiarid sandy region of Northeast China. *Agric. Water Manage.* 243:106414. 10.1016/j.agwat.2020.106414

[B50] Sterk G. Boardman J. Verdoodt A. (2016). Desertification: history, causes and options for its control. *Land Degrad. Dev.* 27 1783–1787. 10.1002/ldr.2525

[B51] Sterner R. W. Elser J. J. (2002). *Ecological Stoichiometry: The Biology of Elements from Molecules to the Biosphere.* Princeton, NJ: Princeton University Press.

[B52] Tian H. Chen G. Zhang C. Melillo J. M. Hall C. A. (2010). Pattern and variation of C:N:P ratios in China’s soil: a synthesis of observational data. *Biogeochemistry* 98 139–151. 10.2307/40647956

[B53] van Heerwaarden L. M. Toet S. Aerts R. (2003). Current measures of nutrient resorption efficiency lead to a substantial underestimation of real resorption efficiency: facts and solutions. *Oikos* 101 664–669. 10.1034/j.1600-0706.2003.12351.x 11841302

[B54] Walbridge M. R. (2000). Phosphorus biogeochemistry. *Ecology* 81 1474–1475. 10.2307/177227

[B55] Wang J. N. Wang J. Y. Wang L. Zhang H. Guo Z. W. Wang G. G. (2019). Does stoichiometric homeostasis differ among tree organs and with tree age? *For. Ecol. Manag.* 453:117637. 10.1016/j.foreco.2019.117637

[B56] Wang K. Song L. N. Lü L. Y. Zhang L. Qin Z. Y. (2014). Fine root biomass vertical distribution character of main afforestation tree species in Horqin Sandy Land. *Bull. Bot. Res.* 34 824–828. 10.7525/j.issn.1673-5102.2014.06.018

[B57] Wang L. L. Li Y. L. Duan Y. L. Lian J. Luo Y. Q. Wang X. Y. (2020). Effects of nitrogen addition and reproductive effort on nutrient resorption of a sand-fixing shrub. *Front. Plant Sci.* 11:588865. 10.3389/fpls.2020.588865 33384703 PMC7769775

[B58] Wang T. (2014). Aeolian desertification and its control in Northern China. *Int. Soil Water Conserv. Res.* 2 34–41. 10.1016/S2095-6339(15)30056-3

[B59] Wang W. J. Lu J. L. Du H. J. Wei C. H. Wang H. M. Fu Y. J. (2017). Ranking thirteen tree species based on their impact on soil physiochemical properties, soil fertility, and carbon sequestration in Northeastern China. *For. Ecol. Manag.* 404 214–229. 10.1016/j.foreco.2017.08.047

[B60] Wojciech P. Ewa B. Jarosław L. Martin L. (2019). A comparison of C:N:P stoichiometry in soil and deadwood at an advanced decomposition stage. *Catena* 179 1–5. 10.1016/j.catena.2019.03.025

[B61] Xiao L. Bi Y. L. Du S. Z. Wang Y. Guo C. Christie P. (2021). Response of ecological stoichiometry and stoichiometric homeostasis in the plant-litter-soil system to re-vegetation type in arid mining subsidence areas. *J. Arid Environ.* 184:104298. 10.1016/j.jaridenv.2020.104298

[B62] Yan T. Lü X. T. Yang K. Zhu J. J. (2016). Leaf nutrient dynamics and nutrient resorption: a comparison between larch plantations and adjacent secondary forests in Northeast China. *J. Plant Ecol.* 9 165–173. 10.1093/jpe/rtv034

[B63] Yan T. Zhu J. J. Yang K. Yu L. Z. Zhang J. X. (2017). Nutrient removal under different harvesting scenarios for larch plantations in northeast China: implications for nutrient conservation and management. *For. Ecol. Manag.* 400 150–158. 10.1016/j.foreco.2017.06.004

[B64] Yang D. X. Song L. Jin G. Z. (2019). The soil C:N:P stoichiometry is more sensitive than the leaf C:N:P stoichiometry to nitrogen addition: a four-year nitrogen addition experiment in a *Pinus koraiensis* plantation. *Plant Soil* 442 183–198. 10.1007/s11104-019-04165-z

[B65] Yang Y. Liu B. R. (2019). Effects of planting *Caragana* shrubs on soil nutrients and stoichiometries in desert steppe of Northwest China. *Catena* 183:104213. 10.1016/j.catena.2019.104213

[B66] Yu Q. Chen Q. S. Elser J. J. He N. P. Wu H. H. Zhang G. M. (2010). Linking stoichiometric homoeostasis with ecosystem structure, functioning and stability. *Ecol. Lett.* 13 1390–1399. 10.1111/j.1461-0248.2010.01532.x 20849443

[B67] Yuan X. B. Niu D. C. Gherardi L. A. Liu Y. B. Wang Y. Elser J. J. (2019). Linkages of stoichiometric imbalances to soil microbial respiration with increasing nitrogen addition: evidence from a long-term grassland experiment. *Soil Biol. Biochem.* 138:107580. 10.1016/j.soilbio.2019.107580

[B68] Zeng D. H. Hu Y. L. Chang S. X. Fan Z. P. (2009). Land cover change effects on soil chemical and biological properties after planting Mongolian pine (*Pinus sylvestris* var. Mongolica) in sandy lands in Keerqin, northeastern China. *Plant Soil* 317 121–133. 10.1007/s11104-008-9793-z

[B69] Zeng D. H. Jiang F. Q. (2006). Deterring “three excesses” (over-cultivation, overgrazing, deforestation) is the only way from the source to control the desertification in ecologically frangible regions in China: taking Keerqin sandy land as an example. *Chin. J. Ecol.* 25 1540–1543. 10.13292/j.1000-4890.2006.0294

[B70] Zhang X. X. Liu Z. W. Zhu Z. H. Du L. Z. (2013). Impacts of decomposition of mixture of leaf litters from *Platycladus orientalis* and other trees on nutrient release. *Acta Pedologica Sin.* 50 181–188.

[B71] Zhao Q. Zeng D. H. Fan Z. P. (2010). Nitrogen and phosphorus transformations in the rhizospheres of three tree species in a nutrient-poor sandy soil. *Appl. Soil Ecol.* 46 341–346. 10.1016/j.apsoil.2010.10.007

[B72] Zhao Q. Zeng D. H. Fan Z. P. Lee D. K. (2008). Effect of land cover change on soil phosphorus fractions in Southeastern Horqin Sandy Land, Northern China. *Pedosphere* 18 741–748. 10.1016/S1002-0160(08)60069-7

[B73] Zhao Q. Zeng D. H. Fan Z. P. Yu Z. Y. Hu Y. L. Zhang J. W. (2009). Seasonal variations in phosphorus fractions in semiarid sandy soils under different vegetation types. *For. Ecol. Manag.* 258 1376–1382. 10.1016/j.foreco.2009.06.047

[B74] Zhou H. H. Chen Y. N. Zhu C. G. Li Z. Fang G. H. Li Y. P. (2020). Climate change may accelerate the decline of desert riparian forest in the lower Tarim River, Northwestern China: evidence from tree-rings of *Populus euphratica*. *Ecol. Indic.* 111:105997. 10.1016/j.ecolind.2019.105997

[B75] Zhu J. J. Li F. Q. Xu M. L. Kang H. Z. Xu D. Y. (2008). The role of ectomycorrhizal fungi in alleviating pine decline in semiarid sandy soil of northern China: an experimental approach. *Ann. For. Sci.* 65:304. 10.1051/forest:2008007

